# Exploratory Study of Predicted Indirectly ReCognizable HLA Epitopes in Mismatched Hematopoietic Cell Transplantations

**DOI:** 10.3389/fimmu.2019.00880

**Published:** 2019-04-24

**Authors:** Kirsten Geneugelijk, Kirsten A. Thus, Hanneke W. M. van Deutekom, Jorg J. A. Calis, Eric Borst, Can Keşmir, Machteld Oudshoorn, Bronno van der Holt, Ellen Meijer, Sacha Zeerleder, Marco R. de Groot, Peter A. von dem Borne, Nicolaas Schaap, Jan Cornelissen, Jürgen Kuball, Eric Spierings

**Affiliations:** ^1^Laboratory of Translational Immunology, University Medical Center Utrecht, Utrecht, Netherlands; ^2^Department of Theoretical Biology and Bioinformatics, Utrecht University, Utrecht, Netherlands; ^3^Matchis Foundation, Leiden, Netherlands; ^4^Department of Immunohematology and Blood Transfusion, Leiden University Medical Center, Leiden, Netherlands; ^5^HOVON Data Center, Department of Hematology, Erasmus MC Cancer Institute, Rotterdam, Netherlands; ^6^Department of Hematology, Cancer Center Amsterdam, Amsterdam UMC, VU Medical Center, Amsterdam, Netherlands; ^7^Department of Hematology, Academic Medical Center, University of Amsterdam, Amsterdam, Netherlands; ^8^Department of Haematology, University of Groningen, University Medical Centre Groningen, Groningen, Netherlands; ^9^Department of Hematology, Leiden University Medical Center, Leiden, Netherlands; ^10^Department of Hematology, Radboud University Medical Center, Nijmegen, Netherlands; ^11^Department of Hematology, Erasmus Medical Center-Daniel Den Hoed Cancer Center, Rotterdam, Netherlands; ^12^Department of Hematology, University Medical Center Utrecht, Utrecht, Netherlands

**Keywords:** HLA, PIRCHE, Non-permissible mismatch, HSCT—hematopoietic stem cell transplant, HLA mismatch

## Abstract

HLA-mismatches in hematopoietic stem-cell transplantation are associated with an impaired overall survival (OS). The aim of this study is to explore whether the Predicted Indirectly ReCognizable HLA-Epitopes (PIRCHE) algorithm can be used to identify HLA-mismatches that are related to an impaired transplant outcome. PIRCHE are computationally predicted peptides derived from the patient's mismatched-HLA molecules that can be presented by donor-patient shared HLA. We retrospectively scored PIRCHE numbers either presented on HLA class-I (PIRCHE-I) or class-II (PIRCHE-II) for a Dutch multicenter cohort of 103 patients who received a single HLA-mismatched (9/10) unrelated donor transplant in an early phase of their disease. These patients were divided into low and high PIRCHE-I and PIRCHE-II groups, based on their PIRCHE scores, and compared using multivariate statistical analysis methods. The high PIRCHE-II group had a significantly impaired OS compared to the low PIRCHE-II group and the 10/10 reference group (HR: 1.86, 95%-CI: 1.02–3.40; and HR: 2.65, 95%-CI: 1.53–4.60, respectively). Overall, PIRCHE-II seem to have a more prominent effect on OS than PIRCHE-I. This impaired OS is probably due to an increased risk for severe acute graft-vs.-host disease. These data suggest that high PIRCHE-II scores may be used to identify non-permissible HLA mismatches within single HLA-mismatched hematopoietic stem-cell transplantations.

## Introduction

HLA mismatching is an important factor in the outcome of allogeneic hematopoietic stem-cell transplantation (HSCT), leading to an impaired overall survival (OS) (1). Therefore, donor and recipient are currently preferably matched for HLA-A, -B, -C, -DRB1, -DQB1 loci (10/10) (2). The detrimental effect of HLA mismatching on OS after HSCT is most pronounced in patients who are transplanted in the early phase of their disease (3), whereas HLA mismatching plays a less prominent role in patients with an advanced disease status before HSCT (3).

HLA-matched (10/10) unrelated donors are available for 23–67% of the patients, depending on the ethnicity (4–6). This underscores the urgent medical need, also in the era of novel transplantation concepts, to discriminate between detrimental HLA mismatches (non-permissible) and well-tolerated HLA (permissible) mismatches. In order to estimate the permissibility of HLA mismatches before transplantation, *in silico* methods can be used to predict whether donor T cells are able to recognize these HLA mismatches (7). We developed recently one of these methods, the so-called “Predicted Indirectly ReCognizable HLA Epitopes” (PIRCHE) algorithm (7–9). The PIRCHE algorithm identifies mismatched HLA-derived epitopes that can potentially be presented on HLA class-I (designated as PIRCHE-I) or HLA class-II molecules (designated as PIRCHE-II). Theoretically, PIRCHE-I lead to CD8+ T-cell responses and PIRCHE-II to CD4+ T-cell responses. The PIRCHE model has shown correlations with transplant outcome in HLA-C and HLA-DPB1 mismatched unrelated donor (MUD) HSCT (8, 9), in HLA-mismatched cord blood transplantation (10), and with *de novo* HLA antibody formation in organ transplantation and pregnancy (11–14). In the current retrospective explorative study we multivariately investigate the role of the PIRCHE algorithm in identifying non-permissible HLA mismatches in 9/10-matched HSCT transplantations.

## Materials and Methods

This study included 685 patients who were transplanted for malignant diseases with MUDs at 8 Dutch transplant centers between 1989 and 2011. A total of 249 patients (36%) were transplanted with a 9/10 match, and 436 (64%) were transplanted with a 10/10 match. Clinical data were collected according to EBMT guidelines (accessible via: https://www.ebmt.org/patient-privacy-statement). All subjects gave informed consent to use their clinical data according to the JACIE guidelines. Since data was collected using the EBMT/JACIE guidelines, additional local ethical approval for conducting the current study was not required in accordance with the institutional requirements and national legislation. The disease status was defined for each individual disease category. Early-stage disease was defined as acute leukemia in first complete remission, chronic myeloid leukemia (CML) in first chronic phase, and myelodysplastic syndrome (MDS), non-Hogdkin lymphoma (NHL), or multiple myeloma (MM) untreated or in first complete remission. Intermediate-stage disease was defined as acute leukemia in second complete remission, CML in second chronic or accelerated phase, MDS in second or partial remission, and NHL or MM in partial remission, second complete remission, or stable disease. Advance-stage disease was defined as acute leukemia, CML, lymphoma, MDS, or MM in later disease stages as defined in de early or intermediate-stage disease. Unambiguous high-resolution HLA-A, -B, -C, -DRB1, and -DQB1 typing data were available for all donor-recipient pairs. HLA typing was performed using sequence-based typing. Supplementary Table 1 shows the population characteristics according to the match status for the complete cohort.

For the 9/10-MUD group, PIRCHE were identified for each donor-recipient pair, as described previously (8, 9). Briefly, NetChop 3.1 (15, 16) (predicting the peptide generation for HLA class-I presentation), and NetMHCpan 2.4 (17, 18) (predicting peptide binding affinity to HLA class-I molecules) were used to identify PIRCHE-I. NetMHCIIPan 3.0 (19, 20) (predicting peptide binding affinity to HLA class-II molecules) was used to identify PIRCHE-II. Only peptides with high binding affinities, i.e., predicted IC_50_ ≤500 nM (for PIRCHE-I) or ≤1,000 nM (for PIRCHE-II), were accepted as relevant binders.

The primary endpoint used in this study was OS, defined as time from HSCT to death due to any cause. To explain the cause of differences in the primary endpoint, several secondary endpoints were evaluated: disease-free survival (DFS; defined as survival without recurrence of the primary malignancy), non-relapse mortality (NRM; defined as mortality without previous progression of the primary malignancy), acute graft-vs.-host-disease (GVHD), chronic GVHD, and progression.

**Figure 1:**
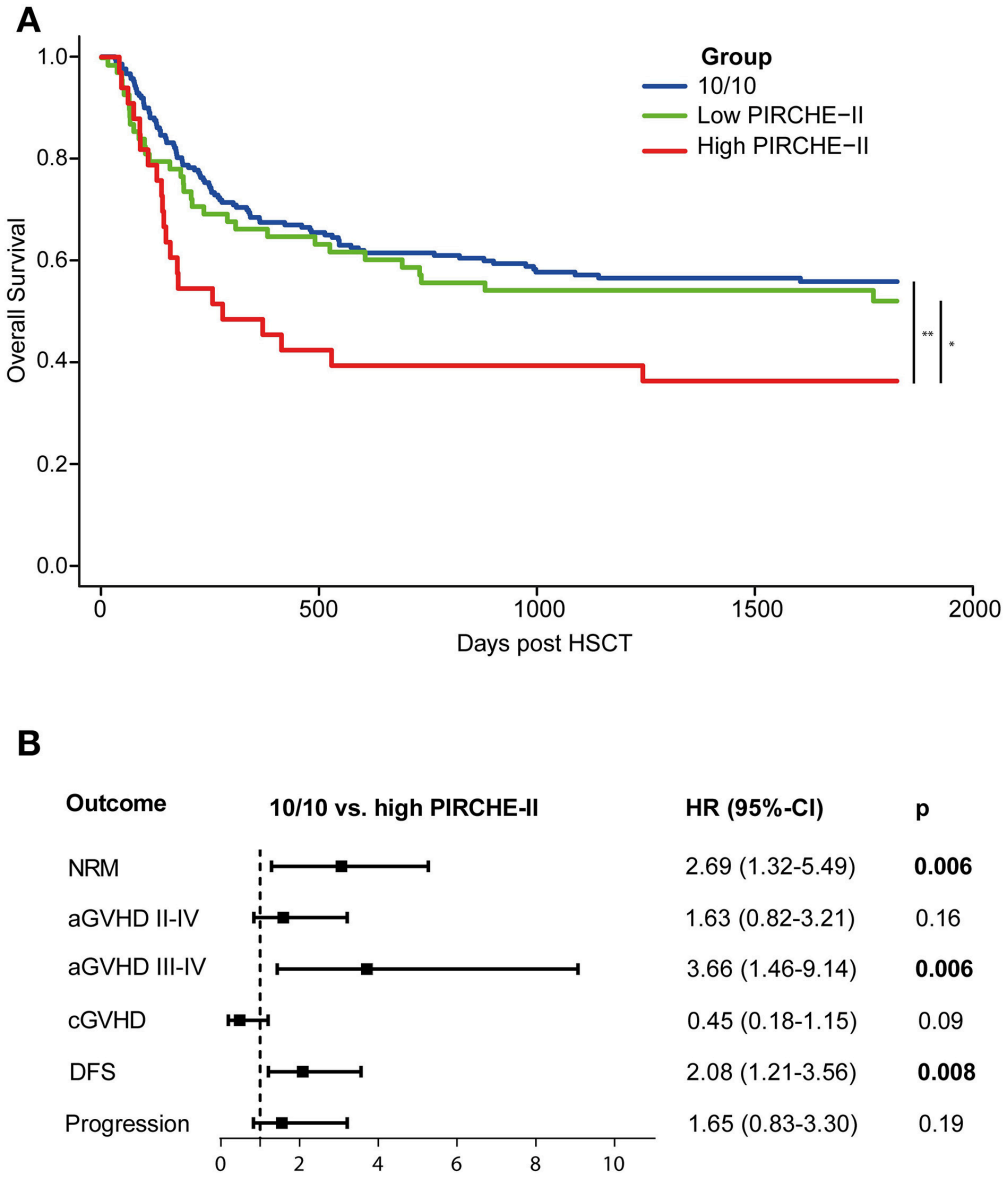
Early-stage disease patients transplanted with a high PIRCHE-II mismatch have an impaired 5-year OS compared to early-stage disease 10/10-matched transplantations **(A)** and a higher risk for severe aGVHD **(B)**. **(A)** High PIRCHE-II had an impaired OS (36%) compared to the 10/10-MUD group (56%) and the low PIRCHE-II group (52%). **(B)** Patients with a high PIRCHE-II mismatch had a significantly increased risk of NRM and acute GVHD compared to 10/10-matched transplantations, whereas the risk for disease progression was not affected. Multivariate models included for NRM: Time to HSCT, KIR ligand status, patient CMV status, conditioning regimen intensity, patient age at transplantation, and donor age; for acute GVHD: donor age (for II-IV) and patient age (for III-IV); for chronic GVHD: aGVHD II-IV, conditioning regimen intensity, patient age at transplantation, stem cell source, and ATG; for DFS: patient age at transplantation; for progression: patient CMV status, conditioning regimen intensity, and ATG. 10/10-MUD: *n* = 212 patients; low PIRCHE-II group: *n* = 70 patients; high PIRCHE-II group: *n* = 33 patients. The number of outcome events in different groups: 10/10: *n* = 94; PIRCHE-II low: *n* = 34; PIRCHE-II high: *n* = 23. OS, overall survival; HR, hazard ratio; 95%-CI, 95%-confidence interval of hazard ratio. PIRCHE-II low, 0–13 PIRCHE-II; PIRCHE-II high, >13 PIRCHE-II; NRM, non-relapse, mortality; GVHD, graft-vs.-host-disease; DFS, disease-free survival.

The association of PIRCHE on OS or DFS was studied with Cox proportional hazard models. Stratification was used to account for heterogeneity of diagnosis. A gamma-frailty term was used to adjust for center effects. Competing risk analyses were performed for NRM (treating progression as a competing risk), acute GVHD, chronic GVHD, and progression. Grading of acute GVHD was defined according to international consensus criteria (21), treating progression and NRM as competing risks. For progression of the primary malignancy, NRM was treated as a competing risk. For all statistical models, the covariates listed in Supplementary Table 1 were evaluated for inclusion in the multivariate models. All statistical analyses were based on multivariate models, except for the Kaplan-Meier analysis (Figure 1A). The Kaplan-Meier analysis was performed univariately to visualize the effect of PIRCHE on OS. Statistical analyses were performed with SPSS 21 (IBM SPSS Software) and with R version 3.4.1 (R Foundation for Statistical Computing). A *p*-value below 0.05 was considered statistically significant.

## Results

The total 9/10-MUD group had significantly impaired OS compared to the 10/10-MUD reference group (Table 1). Since the impact of HLA mismatching on OS highly depends on the disease status before HSCT (3), we subsequently stratified the 9/10-MUD and 10/10-MUD groups according their disease status. Within the early-stage disease patients, 9/10-MUD had significantly impaired OS compared to the 10/10-MUD group [Hazard Ratio (HR): 1.61, 95% confidence interval (95%-CI): 1.09–2.37, *p* = 0.02], whereas for the intermediate and late-stage disease patients no differences in OS were observed between 9/10-MUD and 10/10-MUD (Table 1).

**Table 1:** Hazard ratios of OS for 9/10 compared to 10/10 for different disease stages.

**OS for different groups**			
	**HR**	**95%-CI**	***p***
Overall cohort	1.49	1.14–1.93	0.003
Early disease, *n* = 315	1.61	1.09–2.37	0.02
Intermediate disease, *n* = 229	1.41	0.95–2.08	0.09
Advanced disease, *n* = 100	0.95	0.52–1.74	0.86

*OS, Overall survival; HR, hazard ratio; 95%-CI, 95%-confidence interval of hazard ratio; Multivariate models included for overall cohort: patient age at transplantation, donor age, disease status before transplantation, sex mismatch, patient KIR ligand status, donor CMV, and HSCT year (in three groups); for early disease: patient age at transplantation, donor age, patient KIR ligand status, conditioning regimen intensity, and HSCT year (in three groups); for intermediate disease: patient CMV, donor CMV, and HSCT year (in three groups); for advanced disease: patient age at transplantation, patient KIR ligand status, conditioning regimen intensity, and HSCT year (in three groups)*.

Since our data confirm that the effect of HLA mismatching on OS is more prominent in early-stage disease patients, we next investigated whether PIRCHE scores may aid to identify non-permissible HLA mismatches within this patient group. The baseline characteristics of the early-stage disease patients according to the match grade are listed in Table 2. For the 9/10-MUD group, receiver operating characteristic (ROC) curves with regard to OS were generated for different PIRCHE-I and PIRCHE-II cutoffs (Supplementary Table 2); the cutoffs that yielded the highest area under the curve were used to define low PIRCHE-I/-II and high PIRCHE-I/-II [PIRCHE-I: 0–1 (low) vs. >1 (high); PIRCHE-II: 0–13 (low) vs. >13 (high)]. Statistical comparisons of the co-variates between the 10/10-MUD group and the low or high PIRCHE-I/-II groups showed that only ATG was significantly different between 10/10-MUD and high PIRCHE-I and 10/10-MUD and low PIRCHE-II (data not shown). Both the high PIRCHE-I and the high PIRCHE-II group had a significantly impaired OS compared to the 10/10-MUD early-stage disease group, while only the PIRCHE-II high group had an impaired OS also compared to the PIRCHE-II low group (Table 3). To visualize the effect of PIRCHE-II on OS, univariate Kaplan-Meier analyses were performed (Figure 1A). The high PIRCHE-II group had an impaired 5-year OS (36%) compared to the 10/10-MUD group (56%) and the low PIRCHE-II group (52%). Cumulatively, these data suggest that high PIRCHE-II mismatches are non-permissible HLA mismatches.

**Table 2:** Baseline characteristics of 10/10 and 9/10 groups with an early disease status.

	**10/10,** ***N* (%)**	**9/10,** ***N* (%)**	***P***
Number of patients	212	103	
Patient age at HSCT, median (range)	42 (1–73)	38.5 (1–67)	0.15
Donor age at HSCT, median (range)	34 (19–62)	38 (20–52)	0.05
**Diagnosis**			
Acute leukemia	168	84	0.47
Chronic leukemia	31	15	
Lymphoma	5	0	
Other	7	4	
**Patient sex**			
Male	122 (57.5)	61 (59)	0.77
Female	90 (42.5)	42 (41)	
**Sex mismatch**			
Yes	27 (13)	17 (17)	0.36
No	184 (87)	85 (83)	
**HSCT year**			
1989–1996	14 (7)	10 (10)	0.42
1997–2004	59 (28)	23 (22)	
2004–2011	139 (66)	70 (68)	
**Source**			
BM	70 (33)	36 (35)	0.73
PBSC	142 (67)	67 (65)	
**Conditioning**			
MA	119 (57)	63 (61)	0.45
RIC	91 (43)	40 (39)	
**ATG**			
Yes	95 (45)	63 (61)	0.001
No	117 (55)	36 (35)	
**CMV mismatch**			
Yes	82 (40)	37 (37)	0.53
No	121 (60)	64 (63)	
**C2C2 KIR-ligand status patient**			
Yes	17 (8)	9 (9)	0.83
No	195 (92)	94 (91)	
**EBMT risk score**			
1	33 (16)	18 (18)	0.38
2	42 (20)	29 (28)	
3	82 (39)	36 (35)	
4	50 (24)	17 (17)	
5	4 (2)	2 (2)	
6 or 7	0 (0)	0 (0)	
PIRCHE-I low		49 (48)	
PIRCHE-I high		54 (52)	
PIRCHE-II low		70 (68)	
PIRCHE-II high		33 (32)	

*Differences between the 10/10-MUD and 9/10-MUD group within early-stage disease patients were tested with chi-square for categorical variables and student's T-test for the continuous variable age. HSCT, hematopoietic stem-cell transplantation; Acute leukemia, acute myeloid leukemia (36%); acute lymphoblastic leukemia (24%); myelodysplastic syndrome (30%); other (10%); Chronic leukemia, chronic myeloid leukemia (100%); Lymphoma, non-Hogdkin (100%); Other, multiple myeloma (27%); myeloproliferative neoplasia (72%); PIRCHE-I low, 0–1 PIRCHE-I; PIRCHE-I high, >1 PIRCHE-I; PIRCHE-II low, 0–13 PIRCHE-II; PIRCHE-II high, >13 PIRCHE-II; Sex mismatch, female donor for male patient; BM, bone marrow; PBSC, peripheral blood stem cells; MA, myeloablative; RIC, reduced intensity conditioning; ATG, anti-thymocyte globulin; CMV mismatch, patient seropositive, donor seronegative or patient seronegative, donor seropositive*.

**Table 3:** Hazard ratios of OS for PIRCHE groups compared to 10/10 or to low PIRCHE within the early-stage disease patients.

**PIRCHE groups**	**Compared to 10/10 HSCT**			**Compared to low group**		
	**HR**	**95%-CI**	***p***	**HR**	**95%-CI**	***p***
10/10; *n* = 212	1 (ref)					
PIRCHE-I low; *n* = 49	1.32	0.75–2.30	0.33	1 (ref)		
PIRCHE-I high; *n* = 54	1.86	1.15–2.99	0.01	1.63	0.85–3.11	0.14
10/10; *n* = 212	1 (ref)					
PIRCHE-II low; *n* = 70	1.26	0.79–2.01	0.33	1 (ref)		
PIRCHE-II high; *n* = 33	2.65	1.53–4.60	0.0005	1.86	1.02–3.40	0.04

*OS, Overall survival; HR, hazard ratio; 95%-CI, 95%-confidence interval of hazard ratio; Multivariate models included for PIRCHE groups vs. 10/10-MUD, patient age at transplantation, donor age, HSCT year (in three groups), patient KIR ligand status, and conditioning regimen intensity; for low PIRCHE vs. high PIRCHE: patient age at transplantation, patient KIR ligand status, and conditioning regimen intensity; PIRCHE-I low, 0–1 PIRCHE-I; PIRCHE-I high, >1 PIRCHE-I; PIRCHE-II low, 0–13 PIRCHE-II; PIRCHE-II high, >13 PIRCHE-II; The number of outcome events in different groups, 10/10: n = 94; PIRCHE-I low, n = 23; PIRCHE-I high, n = 34; PIRCHE-II low, n = 34; PIRCHE-II high, n = 23*.

Next, we investigated the potential underlying causes of the impaired OS within the high PIRCHE-II group by studying the secondary endpoints (Figure 1B). Patients transplanted with a non-permissible PIRCHE-II had an increased NRM risk compared to the 10/10-MUD group. Therefore, we investigated potential causes of NRM, such as acute GVHD (22). In our cohort, ~62% of the patients who died of NRM had mild to severe aGVHD. Patients transplanted with a high PIRCHE-II mismatch had a significantly increased risk of severe acute GVHD (grade III-IV) compared to the 10/10-MUD group. In contrast, the risk for chronic GVHD did not differ from the 10/10-MUD group. Since the OS is not only influenced by GVHD, but also by disease progression, we further analyzed the DFS and disease progression risk. Patients transplanted with high PIRCHE-II had an impaired DFS compared to the 10/10-MUD group, whereas no difference in disease progression risk was observed between both groups, suggesting that PIRCHE-II does not affect disease progression. No differences in NRM, GVHD, DFS, and progression risk were observed between the 10/10-MUD group and the low PIRCHE-II group (data not shown), suggesting that low PIRCHE-II scores do not impact alloreactivity and the anti-tumor reactivity.

## Discussion

Several studies have previously shown the association of PIRCHE with alloreactive responses after transplantation. A high number of PIRCHE-II was associated with HLA antibody formation after pregnancy (12) and in different organ transplantation settings (11, 13, 14). Additional studies in cord blood transplantation (10) and in HLA-C and HLA-DPB1 mismatched HSCT (8, 9) have shown that PIRCHE is related with transplant outcome. In the current explorative study we aimed to investigate whether the PIRCHE algorithm can identify non-permissible HLA mismatches in 9/10-matched HSCT transplantations. Since the impact of HLA mismatching on OS is the most prominent in patient transplanted within the early-stage of their disease (3), our analyses were performed within this patient group. Our data suggest that PIRCHE is associated with an impaired overall survival in 9/10-MUD HSCT.

Previous studies in cord blood transplantations have suggested that PIRCHE-II has a higher impact on alloreactivity than PIRCHE-I (10). In pediatric patients, high PIRCHE-I scores were associated with a lower relapse incidence in these patients, whereas a trend for high PIRCHE-II toward an increased GVHD risk was observed (10). Cumulatively, these data suggest that PIRCHE-II might play a more prominent role in GVHD and consequently NRM than PIRCHE-I. Also the current study suggests a more prominent role for PIRCHE-II than for PIRCHE-I. However, we also observed a high association between PIRCHE-I and PIRCHE-II; the majority of the patients with high PIRCHE-II also had high PIRCHE-I (data not shown). When analyzing patients who had both high PIRCHE-I and high PIRCHE-II, this group also had an impaired overall survival compared to the 10/10-MUD HSCT (HR: 2.78; 95%-CI: 1.59–4.86; *p* = 0.0003). This observation suggests that we cannot rule out the effect of PIRCHE-I numbers on the impaired overall survival in the high PIRCHE-II group. Therefore, further validation studies in larger cohorts are warranted to investigate whether PIRCHE-II indeed play a more prominent role in alloreactivity than PIRCHE-I in HLA-mismatched transplantation.

Although our study suggests a more prominent role for PIRCHE-II in alloreactivity than PIRCHE-II, the exact mechanism behind this observation is speculative. Theoretically, high PIRCHE-II numbers are associated with a high level of CD4+ T cell alloreactivity. PIRCHE-II specific CD4+ T cells may impact transplant outcome via two ways [reviewed in (23)]: CD4+ T-helper cells may, after indirect recognition of mismatched HLA, provide help to mismatched HLA-specific CD8+ cytotoxic T cells, and consequently induce alloreactive responses via these CD8+ T cells. On the other hand, PIRCHE-II specific CD4+ T cells may play a role in the formation of HLA-specific antibodies by providing B cell help. A previous study did not show a correlation between engraftment, acute GvHD, and survival after HSCT and the number of mismatched B-cell epitopes between donor and recipient, as predicted by the HLAMatchmaker algorithm (24). Since the previous study did not show a correlation between the number of mismatched B-cell epitopes and survival after mismatched HSCT, it is likely that PIRCHE-II impact overall survival after 9/10-MUD HSCT via providing help to mismatched HLA-specific CD8+ cytotoxic T cells rather than via providing help to B cells.

In the current explorative study we show that PIRCHE is associated with an impaired overall survival in 9/10-MUD HSCT, which was the primary endpoint. The heterogeneity and the small size of the cohort may have biased our results. Consequently, especially the results obtained in the comparison between the low and high PIRCHE group should be treated with caution, as these groups are rather small and the number of events limited. Although a difference in overall survival was observed between low PIRCHE-II and high PIRCHE-II, no differences in NRM, aGVHD, cGVHD, and relapse (the secondary endpoints) were observed between the low and high PIRCHE-II group (data not shown), which may be due to the small group size. Therefore, large (prospective) studies consisting of homogeneous study populations are required to further validate our observations. Moreover, these cohorts are also required to determine the most optimal PIRCHE-II threshold for clinical practice, as the current explorative study does not aim for identifying clinically relevant PIRCHE cutoffs.

In our study we investigated whether the PIRCHE algorithm might identify non-permissible HLA-A, -B, -C, -DRB1, or DQB1 mismatches. The locus mismatches of the early-stage disease 9/10-MUD group within our study cohort are listed in Supplementary Table 3. Individual HLA locus mismatches may have a differential impact on OS (3), which may be due to a differential number of PIRCHE for different locus mismatches. Since our study cohort is rather small, we were unable to investigate the impact of individual HLA locus mismatches on transplant outcome and its' relation with PIRCHE in our cohort.

Although we exclusively focused on HLA-A, -B, -C, -DRB1, and DQB1 mismatches in the current study, compelling evidence indicate that HLA-DPB1 mismatches may impact overall survival after HSCT as well (25, 26). Previously, we have shown in patients transplanted with a 10/10-MUD with a HLA-DPB1 mismatch that HLA-DPB1 mismatches that resulted in a positive PIRCHE score had a higher risk for acute GVHD than those HLA-DPB1 mismatches with a PIRCHE score of zero (8). These data indicate that ideally HLA-DPB1-derived PIRCHE should also be included our analyses. However, HLA-DPB1 typing is lacking for 11% of the study population. Moreover, as the aim of the current explorative study is to study the role of PIRCHE in 9/10 MUD-HSCT, we cannot confirm the previous studies performed in 10/10-MUD HSCT. Nevertheless, our study outcome may have been biased by the occurrence of HLA-DPB1-mismatches among the 9/10-MUD group. The occurrence of HLA-DPB1 mismatches did not significantly differ between the 10/10-MUD and 9/10-MUD group of the early-stage disease patients (*p* = 0.57; data not shown). Also between the low and high PIRCHE group, the occurrence of HLA-DPB1 mismatches did not differ (*p* = 0.31 and *p* = 0.33 for PIRCHE-I and PIRCHE-II, respectively; data not shown), suggesting that our study outcome is likely not biased by the presence of HLA-DPB1 mismatches. When analyzing the number of HLA-DPB1-derived PIRCHE-I and PIRCHE-II, the number of PIRCHE-I and PIRCHE-II derived from HLA-DPB1 did not differ between the low PIRCHE and high PIRCHE group (*p* = 0.56 and *p* = 0.66 for low vs. high PIRCHE-I and low vs. high PIRCHE-II, respectively; data not shown). Nevertheless, further studies in 10/10-MUD HSCT with a HLA-DPB1 mismatch are required to further validate the impact of HLA-DPB1-derived PIRCHE.

The current explorative study focusses on the role of PIRCHE in 9/10 MUD-HSCT. Recently, PIRCHE has been studied in the context of haploidentical HSCT (27). Huo et al. did not find a correlation between PIRCHE and clinical outcome in haploidentical HSCT. The differences in study outcome between this latter study and our current study may (partially) be explained by differences in transplant protocol, in the number of HLA mismatches, and in study design. Moreover, our study confirmed that the impact of HLA matching on OS after HSCT is more prominent in patients who are transplanted in the early phase of their disease. Although our study is restricted to 9/10 MUD-HSCT, the differential impact of HLA mismatching on OS determined by the disease status may also occur in haploidentical HSCT. The analyses in the study of Huo et al. were not stratified according to disease status, which may have impacted the study outcome. Therefore, additional studies are required to further investigate these potential differences between 9/10 MUD-HSCT and haploidentical HSCT.

The PIRCHE calculations are based on the amino acid differences between donor and recipient as well as the ability of shared HLA class I and class II to present epitopes. Therefore, one might argue that the PIRCHE score may just result from the number of amino acid differences between donor and recipient. Since these amino acid differences are one of the underlying factors in the PIRCHE calculations, it is difficult to separate them from the fact that PIRCHE calculations also involve the factor HLA restriction. Therefore, we recalculate the PIRCHE-II scores when scrambling the shared DRB1-background of the patients; i.e., these donor-recipient couples had the same number of amino acid differences, while their presenting HLA-DRB1 allele was scrambled. In these patients, no differences were observed in the OS between the low and high PIRCHE-II group (HR: 1.50; 95%-CI: 0.78–2.90; *p* = 0.29). Furthermore, the impaired OS of the high PIRCHE-II group compared to the 10/10-MUD group was diminished when scrambling the HLA-DRB1 presenting allele (5-years overall survival of 42% for the high PIRCHE-II group). These data indicate that the HLA-DRB1 background of the presenting allele(s) indeed significantly contributes to the observed correlation between PIRCHE-II and OS and that this correlation is not solely the effect of the number of amino acid differences between donor and recipient. These observations are also in line with previous observations in kidney transplantation (11) and in pregnancy (unpublished observations) showing that the correlation of PIRCHE-II with the immunogenicity of HLA mismatches was lost when scrambling the HLA-DRB1 background of the responder.

To identify PIRCHE, we used the NetChop, NetMHCpan, and NetMHCIIpan algorithms. Therefore, the number of PIRCHE highly depends on these algorithms. Although these algorithms are improving over time, prediction of epitope presentation can still be further improved. The prediction of epitope presentation on HLA class II molecules is especially challenging, as HLA class II molecules have a more open binding groove, resulting in a more liberate peptide binding (28, 29). Since epitopes with different lengths can bind to HLA class II molecules, resulting in a different alignment of epitopes in the HLA class II binding groove, the NetMHCIIpan algorithm predicts a core binding motif consisting of nine amino acids (30). Although the prediction of this core binding motif of nine amino acids has been becoming more and more reliable (30, 31), a better prediction of these binding motifs will further improve the PIRCHE algorithm, particularly when estimating specific risks for individual recipient-donor combinations.

The PIRCHE model is not the first model that aims for predicting alloreactivity. However, all these established models have been based on predicting direct T-cell recognition of mismatched HLA, whereas the PIRCHE model predicts indirect T-cell recognition. The Histocheck model (32) has been evaluated in various *in vitro* studies and a large clinical study, but did not correlate with clinical outcome (33–36). Additional studies have shown that the number of amino acid differences within the alpha helices and the beta sheet of HLA class I mismatches are to a certain extend predictive for CD8+ cytotoxic T cell alloreactivity *in vitro* as determined by the CTLp assay (37, 38). HLA class I mismatches that yield more than 5 amino acid differences in the alpha helices and more than 5 amino acid differences in the beta sheet appeared to less immunogenic, whereas HLA class I mismatches that yield less amino acid differences appeared to be highly immunogenic (37, 38). Furthermore, other studies have shown that specific amino acid polymorphisms influence the survival after HSCT (39–42). Additional models based on the physiochemical disparity between HLA class I antigens have shown correlation with transplant outcome; HLA class I mismatches with a high physiochemical disparity are associated with a higher risk of aGVHD after HSCT (43). Moreover, the T-cell epitope (TCE) model for HLA-DP has also shown its effect on HSCT outcome in various studies (44, 45). More recently also an expression model has been evaluated, which showed that the level of cell-surface expression of HLA-DPB1 mismatches is highly predictive for GVHD after HSCT (46, 47). The expression model and the TCE model are highly correlated, suggesting that both models may partially overlap or act synergistically in the prediction of non-permissible HLA-DPB1 mismatches (47). The TCE model may also partly overlap or complement with the PIRCHE model for HLA-DP (8), suggesting that inclusion of both a direct recognition model and an indirect recognition model into one single prediction algorithm may significantly enhance the reliability of the risk classification of specific HLA mismatches.

In conclusion, our data suggest that high PIRCHE-II scores can be classified as non-permissive as these score are associated with an impaired OS after HSCT. These effects on OS are likely due to an increased risk of severe acute GVHD, without affecting progression risk. Our current data suggest that analyzing donors before transplantation for non-permissible PIRCHE-II as part of a donor selection algorithm could be a valuable tool for HLA-mismatched HSCT.

## Ethics Statement

This retrospective study was conducted following the JACIE guidelines. Patients gave informed consent to used their data according JACIE.

## Author Contributions

Contribution: KG, KT, HvD, JC, CK, JK, and ES were involved in design of the work and interpretation of the data. MO, EB, BvdH, EM, SZ, MdG, PvdB, NS, and JC were involved in acquisition of the data. All authors were involved in drafting or revising the manuscript and approved the final version. All authors agree to be accountable for all aspects of the work in ensuring that questions related to the accuracy or integrity of any part of the work are appropriately investigated and resolved.

### Conflict of Interest Statement

The UMC Utrecht has filed a patent application on the prediction of an alloimmune response against mismatched HLA. ES is listed as inventor on this patent. The remaining authors declare that the research was conducted in the absence of any commercial or financial relationships that could be construed as a potential conflict of interest.
