# Supplementary material for: Exploratory Study of Predicted Indirectly ReCognizable HLA Epitopes in Mismatched Hematopoietic Cell Transplantations
Source: Front Immunol. 2019 Apr 24;10:880. doi: 10.3389/fimmu.2019.00880 (PMC6491737; doi:10.3389/fimmu.2019.00880)
Supplement: Supplementary file 1 [file Table_1.DOCX]

|  | **10/10,**  **N(%)** | **9/10,**  **N(%)** | **P** |
| --- | --- | --- | --- |
| Number of patients | 436 | 249 |  |
| Number of pediatric patients | 142 (33) | 50 (20) |  |
| Age at HSCT, median (range) | 42 (0-73) | 40 (0-67) | 0.17 |
| Diagnosis |  |  |  |
| Acute leukemia | 303 (70) | 153 (61) | **0.01** |
| Chronic leukemia | 49 (11) | 24 (10) |  |
| Lymphoma | 60 (14) | 43 (17) |  |
| Other | 24 (6) | 29 (12) |  |
| Patient sex | |  |  |
| Male | 260 (60) | 151 (61) | 0.80 |
| Female | 176 (40) | 98 (39) |  |
| Sex mismatch | |  |  |
| Yes | 59 (14) | 55 (22) | **<0.01** |
| No | 370 (86) | 192 (78) |  |
| HSCT year | |  |  |
| 1989-1996 | 26 (6) | 15 (6) | 0.38 |
| 1997-2004 | 121 (28) | 57 (23) |  |
| 2004-2011 | 290 (67) | 177 (71) |  |
| Source |  |  |  |
| BM | 143 (33) | 76 (31) | 0.54 |
| PBSC | 293 (67) | 173 (70) |  |
| Conditioning | |  |  |
| MA | 227 (52) | 127 (51) | 0.78 |
| RIC | 207 (48) | 121 (49) |  |
| ATG |  |  |  |
| Yes | 232 (53) | 179 (72) | **<0.01** |
| No | 204 (47) | 70 (28) |  |
| Disease status | |  |  |
| Early | 212 (51) | 103 (45) | 0.32 |
| Intermediate | 140 (34) | 89 (39) |  |
| Late | 63 (15) | 37 (16) |  |
| CMV mismatch | |  |  |
| Yes | 167 (39) | 95 (40) | 0.91 |
| No | 258 (61) | 144 (60) |  |
| C2C2 KIR-ligand status patient |  |  |  |
| Yes | 46 (9) | 21 (8) | 0.78 |
| No | 463 (91) | 228 (92) |  |
| EBMT risk score | |  |  |
| 1 | 33 (8) | 18 (8) | 0.05 |
| 2 | 46 (11) | 32 (14) |  |
| 3 | 111 (27) | 53 (23) |  |
| 4 | 107 (26) | 44 (19) |  |
| 5 | 79 (19) | 47 (21) |  |
| 6 or 7 | 32 (8) | 33 (15) |  |
| PIRCHE-I, median (range) |  | 2 (0-17) |  |
| PIRCHE-II, median (range) |  | 7 (0-48) |  |

**Supplementary Table 1: baseline characteristics of 10/10 and 9/10 groups.** Differences between the 10/10 and 9/10 group were tested with chi-square for categorical variables and student’s T test for the continuous variable age. HSCT: hematopoietic stem-cell transplantation. Acute leukemia: acute myeloid leukemia (41%); acute lymphoblastic leukemia (26%); myelodysplastic syndrome (24%); other (8%). Chronic leukemia: chronic myeloid leukemia (100%). Lymphoma: non-Hogdkin (64%), chronic lymphocytic leukemia (26%); Hodgkin (10%). Other: multiple myeloma (57%); myeloproliferative neoplasia (43%). Sex mismatch: female donor for male patient. BM: bone marrow. PBSC: peripheral blood stem cells. MA: myeloablative. RIC: reduced intensity conditioning. ATG: anti-thymocyte globulin. CMV mismatch: patient seropositive, donor seronegative or patient seronegative, donor seropositive.

|  | AUC | P-value |  | AUC | P-value |
| --- | --- | --- | --- | --- | --- |
| PIRCHE-I >0 | 0.570 | 0.22 | PIRCHE-II >0 | 0.512 | 0.84 |
| **PIRCHE-I >1** | **0.581** | **0.16** | PIRCHE-II >1 | 0.547 | 0.42 |
| PIRCHE-I >2 | 0.568 | 0.24 | PIRCHE-II >2 | 0.581 | 0.16 |
| PIRCHE-I >3 | 0.541 | 0.47 | PIRCHE-II >3 | 0.575 | 0.20 |
| PIRCHE-I >4 | 0.497 | 0.96 | PIRCHE-II >4 | 0.577 | 0.18 |
|  |  |  | PIRCHE-II >5 | 0.579 | 0.17 |
|  |  |  | PIRCHE-II >6 | 0.563 | 0.27 |
|  |  |  | PIRCHE-II >7 | 0.557 | 0.33 |
|  |  |  | PIRCHE-II >8 | 0.580 | 0.16 |
|  |  |  | PIRCHE-II >9 | 0.563 | 0.27 |
|  |  |  | PIRCHE-II >10 | 0.556 | 0.32 |
|  |  |  | PIRCHE-II >11 | 0.558 | 0.31 |
|  |  |  | PIRCHE-II >12 | 0.580 | 0.16 |
|  |  |  | **PIRCHE-II >13** | **0.593** | **0.11** |
|  |  |  | PIRCHE-II >14 | 0.584 | 0.14 |
|  |  |  | PIRCHE-II >15 | 0.586 | 0.13 |
|  |  |  | PIRCHE-II >16 | 0.551 | 0.37 |
|  |  |  | PIRCHE-II >17 | 0.534 | 0.56 |
|  |  |  |  |  |  |

**Supplementary Table 2: Assessing the discriminative ability of PIRCHE-I or PIRCHE-II with regard to OS for different PIRCHE-I and PIRCHE-II cutoffs.** AUC: Area under the curve

| **Locus mismatch** | |
| --- | --- |
|  | **n(%)** |
| HLA-A | 21 (20.4) |
| HLA-B | 7 (6.8) |
| HLA-C | 41 (39.8) |
| HLA-DRB1 | 11 (10.7) |
| HLA-DQB1 | 23 (22.3) |
|  |  |

**Supplementary Table 3: locus mismatch in 9/10-MUD early-stage disease patients**
